# Tanshinone IIA Inhibits Epithelial-Mesenchymal Transition in Bladder Cancer Cells via Modulation of STAT3-CCL2 Signaling

**DOI:** 10.3390/ijms18081616

**Published:** 2017-07-25

**Authors:** Sung-Ying Huang, Shu-Fang Chang, Kuan-Fu Liao, Sheng-Chun Chiu

**Affiliations:** 1 Department of Ophthalmology, Hsinchu Mackay Memorial Hospital, No. 690, Sec. 2, Guangfu Rd., East Dist, Hsinchu City 30071, Taiwan; hopes929@gmail.com; 2 Department of Research, Taichung Tzu Chi Hospital, Buddhist Tzu Chi Medical Foundation, No. 88, Section 1, Fengxing Road, Tanzi Dist., Taichung City 427, Taiwan; fantac10@gmail.com; 3 Graduate Institute of Integrated Medicine, China Medical University, No. 91, Hsueh-Shih Road, Taichung City 427, Taiwan; kuanfuliaog@gmail.com; 4 Department of Internal Medicine, Taichung Tzu Chi Hospital, Buddhist Tzu Chi Medical Foundation, No. 88, Section 1, Fengxing Road, Tanzi Dist., Taichung City 427, Taiwan; 5 Department of Laboratory Medicine, Taichung Tzu Chi Hospital, Buddhist Tzu Chi Medical Foundation, No. 88, Section 1, Fengxing Road, Tanzi Dist., Taichung City 427, Taiwan; 6 General Education Center, Tzu Chi University of Science and Technology, No. 880, Section 2, Chien-kuo Road, Hualien City 970, Taiwan

**Keywords:** bladder cancer, chemokine (C-C motif) ligand 2, epithelial-mesenchymal transition, signal transducer and activator of transcription 3, tanshinone IIA

## Abstract

Tanshinone IIA (Tan-IIA) is an extract from the widely used traditional Chinese medicine (TCM) Danshen (*Salvia miltiorrhiza*), and has been found to attenuate the proliferation of bladder cancer (BCa) cells (The IC_50_ were: 5637, 2.6 μg/mL; BFTC, 2 μg/mL; T24, 2.7 μg/mL, respectively.). However, the mechanism of the effect of Tan-IIA on migration inhibition of BCa cells remains unclear. This study investigates the anti-metastatic effect of Tan-IIA in human BCa cells and clarifies its molecular mechanism. Three human BCa cell lines, 5637, BFTC and T24, were used for subsequent experiments. Cell migration and invasion were evaluated by transwell assays. Real-time RT-PCR and western blotting were performed to detect epithelial-mesenchymal transition (EMT)-related gene expression. The enzymatic activity of matrix metalloproteinases (MMP) was evaluated by zymography assay. Tan-IIA inhibited the migration and invasion of human BCa cells. Tan-IIA suppressed both the protein expression and enzymatic activity of MMP-9/-2 in human BCa cells. Tan-IIA up-regulated the epithelial marker E-cadherin and down-regulated mesenchymal markers such as N-cadherin and Vimentin, along with transcription regulators such as Snail and Slug in BCa cells in a time- and dose-dependent manner. Mechanism dissection revealed that Tan-IIA-inhibited BCa cell invasion could function via suppressed chemokine (C-C motif) ligand 2 (CCL2) expression, which could be reversed by the addition of CCL2 recombinant protein. Furthermore, Tan-IIA could inhibit the phosphorylation of the signal transducer and activator of transcription 3 (STAT3) (Tyr705), which cannot be restored by the CCL2 recombinant protein addition. These data implicated that Tan-IIA might suppress EMT on BCa cells through STAT3-CCL2 signaling inhibition. Tan-IIA inhibits EMT of BCa cells via modulation of STAT3-CCL2 signaling. Our findings suggest that Tan-IIA can serve as a potential anti-metastatic agent in BCa therapy.

## 1. Introduction

Bladder cancer (BCa) is one of the most prevalent types of cancer and is the leading cause of death among patients with urinary tract disease [1]. In 2016, the United States alone recorded more than 76,000 new cases of BCa and 16,000 deaths [2]. Most BCa cases are diagnosed as non-muscle invasive tumors; however, 50–70% of these tumors recur frequently and approximately 15% eventually develop into muscle-invasive or metastatic BCa [3,4]. Current treatment methods including radical cystectomy and systemic chemotherapy are effective in some muscle-invasive BCa patients, but 95% of metastatic BCa patients die within 5-years diagnosis, indicating the need for new therapeutic strategies [5].

Tan-IIA (C_19_H_18_O_3_) is one of the major lipophilic compounds extracted from the root of a traditional Chinese medicine, Danshen (*Salvia miltiorrhiza*) [6,7], and has been used for the treatment of cardiovascular disease via its anti-oxidant and anti-inflammatory activity [8,9]. In addition, Tan-IIA has been found to exert antitumor activity in various types of cancer including osteosarcoma [10], gastric [11], lung [12], esophageal [13], and prostate cancers [14]. The antitumor activity of Tan-IIA mainly occurs through proliferation inhibition, apoptosis induction, and metastasis inhibition [15,16,17,18]. For instance, Tan-IIA increased CCAAT/enhancer-binding protein homologous protein (CHOP) and caspase-4 expression, and induced apoptosis of human esophageal Ec-109 cells via the endoplasmic reticulum (ER) stress pathway [19]. Tan-IIA induced cytochrome c-mediated caspase cascade apoptosis in A549 human lung cancer cells via the JNK pathway [20]. Tan-IIA caused apoptosis in human oral cancer KB cells through a mitochondria-dependent pathway [21]. However, Tan-IIA did not show significant cytotoxicity on human normal prostate epithelial cells (PrEC) and normal mammary epithelial cells (HMEC) at the concentrations high as 50 μM [22,23]. Also, the toxicity in normal tissues was not observed in Tan-IIA treated mice [24]. In our previous study, Tan-IIA was found to induce mitochondria-dependent apoptosis and suppress migration in BCa cells [25]. However, the mechanism by which Tan-IIA inhibits the migration and invasion of BCa cells remains undetermined.

Previous reports found a correlation of urinary CCL2 levels with tumor stage, grade and metastasis in patients with BCa [26,27], and patients with stages T2–T4 BCa were found to have a higher mean CCL2 concentration in their urine as compared to those with T1 stage tumors [27]. Previous studies also showed that CCL2 can regulate tumor progression and metastasis by altering the tumor microenvironment [28,29,30]. CCL2 induced epithelial mesenchymal transition (EMT) in order to promote tumor metastasis in various cancer types [31,32,33]. Down-regulation of CCL2 expression by inhibiting phosphorylation of STAT3 led to the suppression of metastasis in breast and lung cancer [34]. STAT3 signaling is an important pathway which is frequently activated in many tumors including BCa [35,36]. The transcriptional activity of STAT3 is required for the phosphorylation at the tyrosine residue 705 (Tyr705) and has been demonstrated to be critical for BCa cell growth and survival [36,37]. In addition, activation of STAT3 promoted migration and invasion of BCa cells [38]. Thus, we seek to elucidate the role of STAT3-CCL2 signaling in Tan-IIA-induced EMT inhibition in BCa cells.

The results of the present study demonstrate that Tan-IIA inhibited the migration and invasion of human BCa cells. Tan-IIA inhibited EMT in BCa cells via the suppression of CCL2 expression which cannot be reversed by addition of CCL2 recombinant protein. In addition, Tan-IIA suppressed the phosphorylation of STAT3 (Tyr705), which cannot be restored by addition of CCL2 recombinant protein. Our data suggests that Tan-IIA might inhibit EMT in BCa cells through the STAT3-CCL2 signaling inhibition.

## 2. Results

### 2.1. Tan-IIA Inhibits the Migration and Invasion of Human BCa Cells

**Figure 1:**
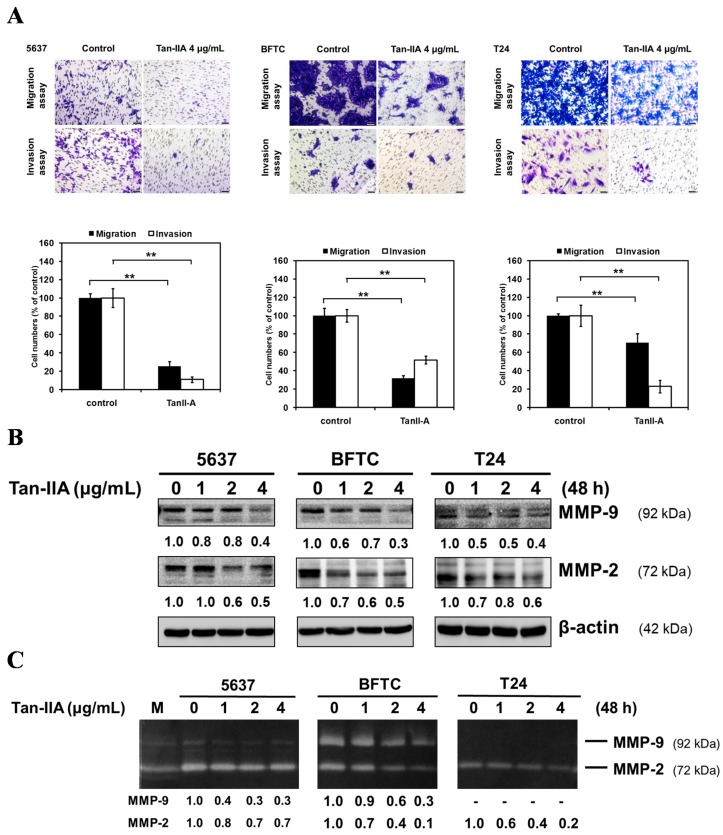
Tan-IIA inhibited migratory and invasive ability in human BCa cells. (**A**) Human BCa cells were treated with 0.2% DMSO as a vehicle control or 4 μg/mL Tan-IIA for 24 h and then seeded onto the transwell hanging insert for migration (24 h) and invasion (48 h) assays. Images were captured using an inverted microscope with 200× magnification; Scale bar: 50 μm. The migration and invasion of BCa cells were quantified by counting the stained cells that migrated into the underside of the hanging insert membrane; (**B**) human BCa cells were treated with different concentrations of Tan-IIA (1, 2 and 4 μg/mL) for 48 h. The protein of total cell lysates were then used to detect MMP-9/-2 protein expression using western blot, and the (**C**) supernatant was used to detect the enzymatic activity using zymography analysis. M: marker. Data are presented as means ± S.D. from three different experiments. ** *p* < 0.01 versus vehicle.

Human BCa cells were treated with 4 μg/mL Tan-IIA for 24 h and then subjected to migration (24 h) and invasion (48 h) assay (Figure 1A). In the migration assay, Tan-IIA decreased the number of migrating cells to 25.6 ± 4.7% (5637), 32 ± 2.9% (BFTC), and 70.5 ± 9.7% (T24) as compared to the control group. In the invasion assay, Tan-IIA decreased the number of migrating cells to 11 ± 2.9% (5637), 51.8 ± 4.4% (BFTC), and 22.8 ± 9.8% (T24) as compared to the control group.

Western blot results indicated Tan-IIA down-regulated the protein expression of MMP-9/-2 in a dose-dependent manner (Figure 1B). Zymography analysis also showed that Tan-IIA attenuated the enzymatic activity of MMP-9/-2 in a dose-dependent manner (Figure 1C). Taken together, these results suggested that Tan-IIA might be an effective inhibitor of cell migration and invasion of BCa cells.

### 2.2. Tan-IIA Inhibits EMT in Human BCa Cells

**Figure 2:**
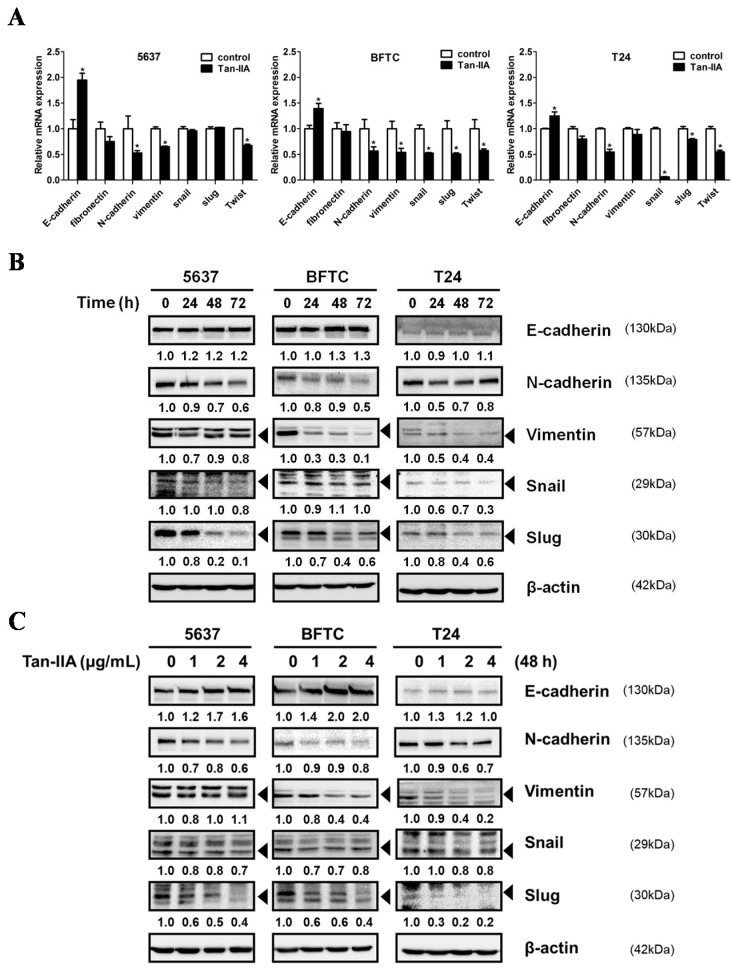
Tan-IIA inhibited EMT on human BCa cells. (**A**) Human BCa cells were treated with 4 μg/mL Tan-IIA for 24 h. The expression of EMT-related genes was detected by qRT-PCR analysis; (**B**) human BCa cells were treated with 4 μg/mL Tan-IIA for 24 to 72 h. The expressions of EMT-related genes were detected by western blot. (**C**) Human BCa cells were treated with increasing concentrations of Tan-IIA (1, 2 and 4 μg/mL) for 48 h. The expressions of EMT-related genes were detected by western blot. Data are presented as means ± S.D. from three different experiments. * *p* < 0.05 versus vehicle.

EMT is a crucial step for the invasion and metastasis of BCa cells. We first show that Tan-IIA could inhibit cellular migration and invasion in BCa cells, and this is accompanied by the up-regulation of epithelial marker E-cadherin, the down-regulation of mesenchymal markers N-cadherin and Vimentin, and the down-regulation of transcription factor Snail and Slug, at both the mRNA and protein level as evidenced by quantitative RT-PCR (qRT-PCR) (Figure 2A) and western blot (Figure 2B,C).

### 2.3. Tan-IIA Inhibits EMT via Down-Regulated CCL2 Expression in Human BCa Cells

**Figure 3:**
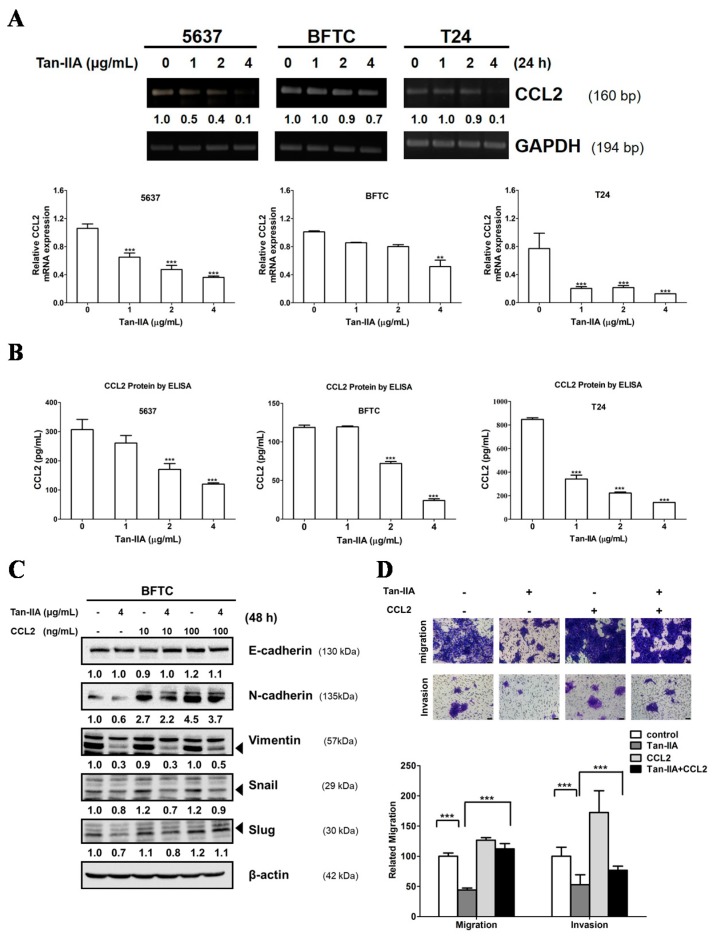
Tan-IIA inhibited the CCL2 expression and reversed the EMT in human BCa cells. (**A**) Human BCa cells were treated with increasing concentrations of Tan-IIA for 24 h. The expression of CCL2 was detected by PCR and qRT-PCR; (**B**) Human BCa cells were treated with increasing concentrations of Tan-IIA for 48 h. The supernatant was collected for CCL2 protein detection using ELISA assay; (**C**) BFTC cells were treated with or without 4 μg/mL Tan-IIA in the presence or absence of CCL2 recombinant protein for 48 h. The EMT-related gene expression was detected by western blot; (**D**) BFTC cells were treated with or without 4 μg/mL Tan-IIA in the presence or absence of 100 ng/mL human CCL2 recombinant protein for 24 h, followed by migration (24 h) or invasion (48 h) assays and analyzed as previous described. Data are presented as means ± S.D. from three different experiments. *** *p* < 0.001 versus vehicle.

Previous reports suggested that high levels of CCL2 expression play a key role in BCa progression and metastasis in vitro and in vivo [27,32,39]. Thus, we analyzed the CCL2 expression in the culture medium of human BCa cells treated with or without Tan-IIA. As shown in Figure 3A, Tan-IIA inhibited the CCL2 expression in all BCa cell lines detected by PCR and qRT-PCR. Furthermore, ELISA tests confirmed that the protein level of CCL2 secreted by BCa cells was inhibited by Tan-IIA treatment in a dose-dependent manner (Figure 3B). These results showed that Tan-IIA down-regulated CCL2 expression in BCa cells.

To investigate the mechanism by which Tan-IIA inhibits CCL2 resulting in metastatic inhibition, BFTC cells were treated with or without human CCL2 recombinant protein (10 or 100 ng/mL) in the presence or absence of 4 μg/mL Tan-IIA for 48 h to examine the EMT-related genes expression. As shown in Figure 3C, treatment with CCL2 recombinant protein increased the expression of mesenchymal marker N-cadherin and Vimentin, along with transcription factor Snail and Slug, which were down-regulated by Tan-IIA treatment. In addition, treatment with CCL2 recombinant protein attenuated the inhibitory effect on migration and invasion induced by Tan-IIA treatment (Figure 3D). Together, these findings indicate that Tan-IIA inhibited EMT in BCa cells via the down-regulation of CCL2.

### 2.4. Tan-IIA Inhibits STAT3-CCL2 Signaling in Human BCa Cells

**Figure 4:**
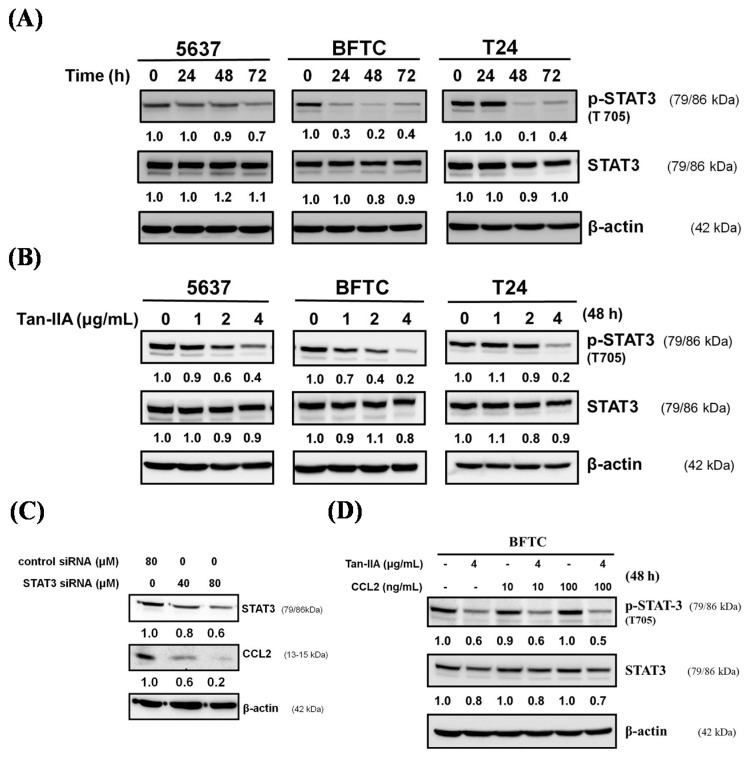
Tan-IIA inhibited STAT3-CCL2 signaling in human BCa cells. (**A**) Human BCa cells were treated with 4 μg/mL Tan-IIA for indicated time points, and the expression of phospho-STAT3 (T705) was detected by western blot; (**B**) human BCa cells were treated with increasing concentrations of Tan-IIA for 48 h, the expression of phospho-STAT3 (T705) were detected by western blot; (**C**) BFTC cells were transfected with control or STAT3 siRNA for 24 h, and the expression of STAT3 and CCL2 was detected by western blot; (**D**) BFTC cells were treated with or without 4 μg/mL Tan-IIA in the presence or absence of human CCL2 recombinant protein for 48 h. The expression of phospho-STAT3 (T705) and STAT3 was detected by western blot.

Recent studies indicated that CCL2 signaling plays a pivotal role in regulating STAT3 activation and EMT [40], and the inhibition of STAT3 signaling may reduce the invasiveness of BCa [41]. We further examined whether Tan-IIA could inhibit the activation of STAT3 on BCa cells. Human BCa cells were treated with Tan-IIA for indicated time points and p-STAT3 (Tyr705) was analyzed by western blot. As shown in Figure 4A,B, Tan-IIA inhibited the activation of STAT3 by decreasing the phosphorylation of STAT3 at Tyr705 in all BCa cell lines in a time- and dose-dependent manner. To elucidate the mechanism by which Tan-IIA inhibits CCL2 through regulating STAT3, BFTC cells were transfected with the STAT3 siRNA and the expression of STAT3 and CCL2 were examined by western blot. Silencing the expression of STAT3 leads to the inhibition of CCL2 expression (Figure 4C). However, treatment with human CCL2 recombinant protein (10 or 100 ng/mL) cannot restore the regulation of STAT3 via phosphorylation of Tyr705, and this was inhibited by Tan-IIA treatment. These results suggested that Tan-IIA down-regulated the CCL2 expression via inhibition of the STAT3 pathway in human BCa cells.

## 3. Discussion

Our previous study reported that Tan-IIA could inhibit the proliferation and migration of human BCa cells [25], but the underlying mechanism of Tan-IIA attenuating the migration and invasion of BCa cells remains unclear. EMT is a process by which epithelial cells gradually transform into mesenchymal-like cells to promote the migration and invasiveness of cancer cells [42]. Our results showed that Tan-IIA treatment could inhibit the process of EMT as evidenced by increased level of the epithelial marker E-cadherin and decreased level of mesenchymal markers (N-cadherin and Vimentin). Activation of MMP proteins leads to cell migration and penetration to the basement membrane, playing an important role in EMT processes [43]. In previous studies, Tan-IIA decreased migration or invasion through inhibiting MMP-9/-2 secretion in gastric cancer and osteosarcoma [11,18]. Similar results observed in our study showed that Tan-IIA suppressed both the protein expression and enzymatic activity of MMP-9/-2 on human BCa cells (Figure 1B). Together, these findings suggest that Tan-IIA inhibits EMT in human BCa cells.

Several reports have demonstrated the importance of CCL2 and EMT signals in BCa progression. Chiu et al. reported that blocking the CCL2/CCR2 pathway could decrease the migration and invasion of BCa cells [39]. Additional reports show that CCL2 signals promote EMT in various tumors including BCa [32,40,44,45]. The present study provides evidence that Tan-IIA decreased CCL2 expression in a dose-dependent manner by qRT-PCR and ELISA analysis (Figure 3). The addition of CCL2 recombinant protein resulted in a partial reversal of EMT markers, and attenuated the Tan-IIA-induced migration and invasion inhibition in BCa cells. Our results show that Tan-IIA inhibits the EMT in BCa cells via the suppression of CCL2 expression.

**Figure 5:**
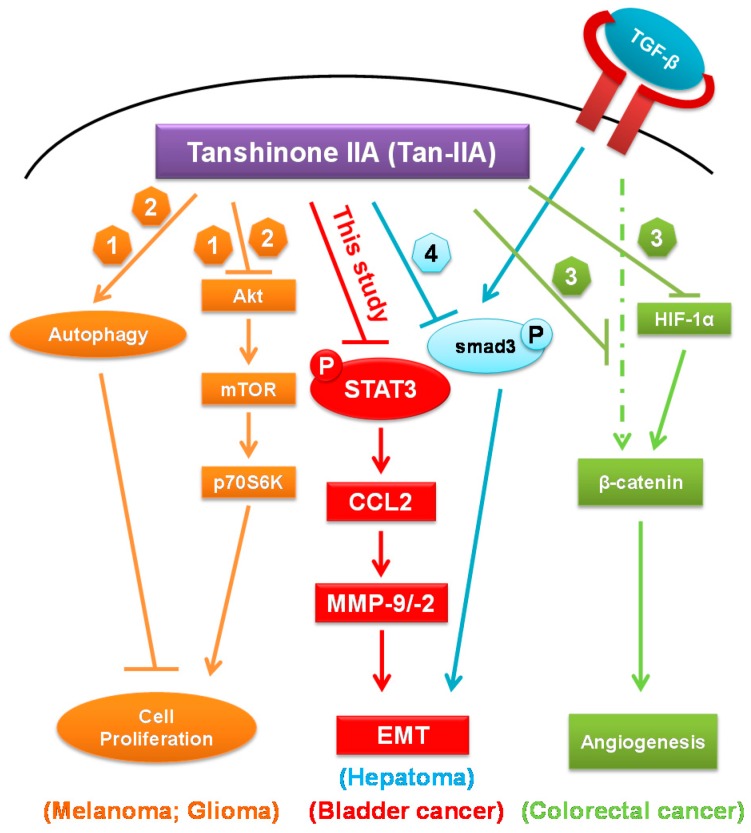
Schematic representation the anti-cancerogenic roles of Tan-IIA. CCL2: Chemokine (C-C motif) ligand 2; EMT: Epithelial-Mesenchymal Transition; HIF-1α: Hypoxia-inducible factor-1α; MMP: Matrix MetalloProteinases; mTOR: mammalian target of rapamycin; P: phosphorylation; p70S6K: p70 ribosomal protein S6 kinase; STAT3: signal transducer and activator of transcription 3; Tan-IIA: Tanshinone IIA; TGF-β: transforming growth factor β. ↓: stimulatory modification; ⊥: inhibitory modification; Dashed arrow: putative stimulatory modification [47,48,49,50].

Additional reported data suggested that CCL2 induced EMT through the activation of STAT3 signals [33,40] and inhibited STAT3 signaling to reduce the invasiveness of tumor cells [41,46]. Our data showed that Tan-IIA could inhibit the p-STAT3 (Tyr705) in a time- and dose-dependent manner. Besides, inhibition of STAT3 expression by STAT3 siRNA transfection attenuated the expression of CCL2. The phosphorylation of STAT3, inhibited by Tan-IIA, cannot be restored by CCL2 recombinant protein addition. These data suggested that Tan-IIA inhibits EMT of human BCa cells via modulation of STAT3-CCL2 signaling (Figure 5). Several effects of Tan-IIA on human cancer were also integrated to get a better view of possible anti-cancerogenic effects of Tan-IIA [47,48,49,50]. In addition, since the results from this study were based on in vitro assays of human BCa cells, the in vivo experiments are necessary for future study.

EMT is orchestrated by several signaling pathways, including JAK/STAT3 and TGF-β/Smad signaling. Recent studies have demonstrated that TGF-β-mediated cancer metastasis was associated with the activation of STAT3 pathway in colorectal and lung cancer [51,52]. STAT3 activation can increase smad7 expression and form an inhibitory complex with smad3 which eventually suppress EMT [53]. Recent study elucidated the mechanisms behind the tumoricidal activity of TCM clinical prescription Jianpi Huayu Decoction (JHD) in Hepatocellular carcinoma (HCC) treatment. Their results indicated that Tan-IIA might be the one of crucial components of the JHD that targets on the TGF-β/Smad3 pathway and inhibits EMT [50]. Taken together, the targeted blockade of the STAT3/smad3 axis in tumor cells may be an effective therapeutic strategy against tumor metastatic progression and worth for further investigation.

## 4. Materials and Methods

### 4.1. Chemicals and Antibodies

Tanshinone IIA (C_19_H_18_O_3_, >97% HPLC), Dimethyl sulfoxide (DMSO), [3-(4,5-dimethyl thizol-2-yl)-2,5-diphenyl tetrazolium bromide] (MTT), Tween-20, methanol, and horseradish peroxidase-conjugated secondary antibodies were purchased from Sigma Chemical Co. (St. Louis, MO, USA). The antibodies against p-STAT3 (Tyr705), STAT3, E-cadherin, N-cadherin, Vimentin, Slug, Snail, MMP-2, MMP-9 and β-actin were all purchased from Cell Signaling Technology, Inc., (Danvers, MA, USA). The human CCL2 recombinant protein was purchased from Santa Cruz Biotechnology, Inc. (Dallas, TX, USA). Polyvinyldenefluoride (PVDF) membranes, BSA protein assay kit and western blot chemiluminescence reagent were purchased from Amersham Biosciences (Arlington Heights, IL, USA).

### 4.2. Cell Culture

The human BCa cell lines 5637 (grade II carcinoma), BFTC (BFTC 905, papillary transitional cell carcinoma), and T24 (transitional cell carcinoma) were purchased from BCRC (Bioresource Collection and Research Center, Hsinchu, Taiwan). Cells were cultured in appropriate medium supplemented with 10% FBS, 100 U/mL penicillin and 100 U/mL streptomycin (all from Invitrogen, Carlsbad, CA, USA) at 37 °C in a humidified atmosphere with 5% CO_2_.

### 4.3. Western Blot Analysis

Five hundred thousand cells per 6-cm plate were lysed with 200 µL M-PER mammalian protein extraction reagent containing protease inhibitor cocktail (Thermo Scientific, Rockford, IL, USA) and centrifuged at 13,000× *g* at 4 °C for 10 min. The protein concentration in the supernatants was quantified using a BSA Protein Assay Kit. Electrophoresis was performed on a NuPAGE Bis-Tris Electrophoresis System using 20 µg of reduced protein extract per lane. Resolved proteins were transferred to PVDF membranes, blocked with 5% skim milk for 1 h at room temperature, finally probed with the specific primary antibodies at 4 °C overnight. After the PVDF membrane was washed three times with TBS/0.2% Tween-20 at room temperature, it was incubated with appropriate secondary antibody labeled with horseradish peroxidase (Sigma Chemical, St. Louis, MO, USA) for 1 h at room temperature. All resolved proteins bands were detected using Western Lightning™ Chemiluminescence Reagent Plus (Amersham Biosciences, Arlington Heights, IL, USA).

### 4.4. Cell Migration and Invasion Assay

The trans-well assay was performed using Hanging inserts (Millipore Co., Billerica, MA, USA) with 8 μm polycarbonate membrane in a 24-well plate. Cells were seeded in 6 well plates and treated without or with 4 μg/mL Tan-IIA with or without CCL2 for 24 h. Cells were then detached and seeded (5 × 10^4^) to the upper chamber of the transwell plates. Upper chambers were filled with serum free medium and lower chambers were filled with cultured medium containing 10% FBS as a chemo-attractant. Incubation was carried out at 37 °C for the indicated 24 h. The hanging inserts were washed with PBS, and cells on the upper filter surface were wiped away with a cotton swab. The inserts were subsequently fixed with 10% formalin for 10 min at room temperature, stained with 0.2% *w*/*v* crystal violet, washed with PBS, the remaining cells were counted on the opposite site of the filter under a light microscope operating at 200× magnification. The migration cell numbers of control group were considered as 100%. For the invasion assay, a Matrigel basement membrane matrix (BD Biosciences, San Jose, CA, USA) was coated to the upper side of the hanging inserts at a concentration of 2 mg/mL. Cells were seeded onto the coated hanging inserts and followed by migration assay protocol.

### 4.5. RNA Extraction and Real-Time RT-PCR

Total RNA was extracted from cell lines using RNeasy Mini Kit^®^ (Qiagen, Valencia, CA, USA) and reverse transcribed at 37 °C for 60 min with Omniscript RT Kit^®^ (Qiagen) according to the manufacturer’s instructions. Real-time RT-PCR analysis was performed in triplicate in a Step One Plus Real-Time PCR system (Applied Biosystems, Foster City, CA, USA) with Power SYBR^®^ Green PCR Master Mix (Applied Biosystems) in a final volume of 20 μL/reaction. Threshold cycle (*C*_t_) value of each tested gene was normalized to the *C*_t_ value of the GAPDH control from the same RNA preparation. The ratio of transcription of each gene was calculated as 2^–(Δ*C*t)^, where Δ*C*_t_ is the difference *C*_t_(test gene)−*C*_t_(GAPDH). Real-time RT-PCR primer sequences used in this study are listed in Table 1.

### 4.6. Enzyme-Linked Immunosorbent Assay (ELISA)

Human MCP-1/CCL2 ELISA kit was purchased from R&D Systems. BCa cells were cultured in serum-free medium with or without Tan-IIA for 72 h. The medium were collected (400 μL/sample in 96-well) for ELISA assay according to manufacturer’s instructions.

### 4.7. Gelatin Zymography

The BCa cells were cultured in serum-free medium containing Tan-IIA (0, 1, 2, 4 μg/mL) for 48 h and the supernatant was collected. The supernatant was mixed with non-reducing SDS gel sample buffer. Electrophoresis was carried out using 10% native polyacrylamide gel containing 0.1% gelatin (Sigma, St. Louis, MO, USA) on a NuPAGE Bis-Tris Electrophoresis System. After electrophoresis, the gels were washed in wash buffer containing 2.5% Triton X-100 at room temperature, and then incubated with the reaction buffer containing l M CaC1_2_, 2% NaN_3_, 1 M Tris-HCl (pH 8.0) at 37 °C overnight. Gels were stained by Coomassie Brilliant Blue R-250 solution and gelatinolytic activity was shown as clear areas in the gel.

### 4.8. Small Interfering RNA (siRNA) Transfection

STAT3 siRNA (#6582) was purchased from Cell Signaling Technology, Inc., (Danvers, MA, USA). Non-targeting siRNA (ON-TARGET plus non-targeting pool) were purchased from Dharmacon RNAi Technologies (Lafayette, CO, USA). Non-targeting control sequences were not provided. BFTC cells at 50–60% confluence were transfected with siRNA (40 or 80 nM) using the DharmaFECT 4 transfection reagents (GE Healthcare Dharmacon, Lafayette, CO, USA) according to the manufacturer’s protocol. Cells were cultured for 24 h, and then treated with Tan-IIA or vehicle for an additional 48 h. Proteins were then isolated for western blotting.

### 4.9. Statistical Analysis

All data were shown as mean ± S.D. Statistical differences were analyzed using the Student’s *t*-test for normally distributed values.

## 5. Conclusions

In conclusion, our study demonstrated that Tan-IIA inhibits EMT in human BCa cells. The anti-metastatic effects of Tan-IIA in human BCa cells were shown by migration and invasion assay. Tan-IIA is shown to regulate EMT-related gene expression via the suppression of CCL2. The inhibition of CCL2 might be linked to the phosphorylation inhibition at Tyr705 of STAT3 by Tan-IIA. Tan-IIA has been shown to inhibit EMT in human BCa cells, and the mechanism involved was mediated through the modulation of STAT3-CCL2 signaling. Thus, our findings suggest a novel role of Tan-IIA in controlling BCa, suggesting that Tan-IIA might be a potential option for treating BCa metastasis.

## Figures and Tables

**Table 1:** The gene-specific primers used in this study.

Gene	Primers
*CCL2*	sense: 5′-GATCTCAGTGCAGAGGCTCG-3′ antisense: 5′-TGCTTGTCCAGGTGGTCCAT-3′
*E-cadherin*	sense: 5′-ACGTCGTAATCACCACACTGA-3′ antisense: 5′-TTCGTCACTGCTACGTGTAGAA-3′
*N-cadherin*	sense: 5′-ACAGTGGCCACCTACAAAGG-3′ antisense: 5′-CCGAGATGGGGTTGATAATG-3′
*Fibronectin*	sense: 5′-CCCACCGTCTCAACATGCTTAG-3′ antisense: 5′-CTCGGCTTCCTCCATAACAAGTAC-3′
*Vimentin*	sense: 5′-CTTCGCCAACTACATCGACA-3′ antisense: 5′-GCTTCAACGGCAAAGTTCTC-3′
*Snail*	sense: 5′-TCGTCCTTCTCCTCTACTTC-3′ antisense: 5′-TTCCTTGTTGCAGTATTTGC-3′
*Slug*	sense: 5′-TGTTGCAGTGAGGGCAAGAA-3′ antisense: 5′-GACCCTGGTTGCTTCAAGGA-3′
*GAPDH*	sense: 5′-CCATGGAGAAGGCTGGGG-3′ antisense: 5′-CAAAGTTGTCATGGATGACC-3′

## Abbreviations

CCL2	Chemokine (C-C motif) ligand 2
EMT	Epithelial-mesenchymal transition
MMP	Matrix metalloproteinases
STAT3	Signal transducer and activator of transcription 3
Tan-IIA	Tanshinone IIA
